# Enhancing Chitin Production as a Fermentation Byproduct through a Genetic Toolbox That Activates the Cell Wall Integrity Response

**DOI:** 10.1021/acssynbio.4c00436

**Published:** 2025-01-06

**Authors:** An Nguyen, Isabell Tunn, Merja Penttilä, Alexander D. Frey

**Affiliations:** Department of Bioproducts and Biosystems, Aalto University, Espoo 02150, Finland

**Keywords:** coproduction, cell wall integrity pathway, chitin, RHO1, PKC1, Saccharomyces cerevisiae

## Abstract

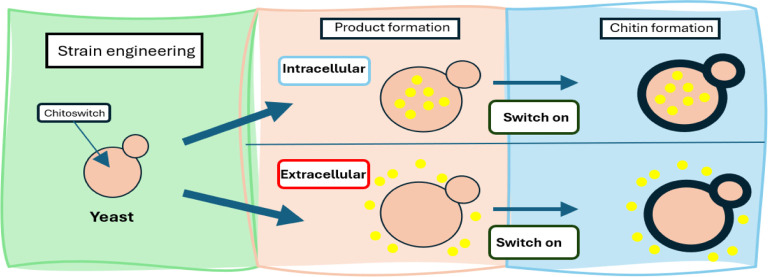

Often, the value of the whole biomass from fermentation processes is not exploited, as commercial interests are focused on the main product that is typically either accumulated within cells or secreted into the medium. One underutilized fraction of yeast cells is the cell wall that contains valuable polysaccharides, such as chitin, known for its biocompatibility and biodegradability, which are thought of as valuable properties in diverse industries. Therefore, the valorization of waste biomass from fermentation to coproduce chitin could significantly improve the overall profitability and sustainability of biomanufacturing processes. Previous studies revealed that environmental stresses trigger the cell wall integrity (CWI) response, leading to an increased level of chitin synthesis as a protective measure. In this study, we evaluated the use of the key regulatory genes of the CWI response, *RHO1* and *PKC1,* and their mutant forms *RHO1*^*Q68H*^*and PKC1*^*R398A*^, to design a genetic switch that provides control over the CWI response to maximize the chitin content in the cell wall. The generated genetic control elements were introduced into different yeast strains, among others, for the coproduction of chitin with either storage lipids or recombinant proteins. Overall, we successfully increased the chitin content in the yeast cell wall up to five times with our optimized setup. Furthermore, similar improvements in chitin production were seen when coproducing chitin with either storage lipids or a secreted acid phosphatase. Our results successfully demonstrated the potential of maximizing the chitin content in the cell wall fraction while producing other intra- or extracellular compounds, showcasing a promising approach for enhancing the efficiency and sustainability of fermentation processes. Moreover, the chitin produced in the cell wall is indistinguishable from the chitin isolated from crustaceans.

## 1 Introduction

Products manufactured using fermentation processes are often expensive as a large fraction of the produced cell mass is not used or utilized as a low-value byproduct such as feed.^1^ A particular fraction of the waste biomass with a potential commercial value is the fungal cell wall, as it contains a range of polysaccharides with interesting and chemically different properties. Among those substances is chitin, a rigid polysaccharide composed of β-1,4-linked *N*-acetyl-β-d-glucosamine (GlcNAc) that is biocompatible and biodegradable, and exhibits excellent absorption and antimicrobial properties.^2^ As a result, it is considered as a valuable starting material for applications in many different fields such as agriculture, wastewater treatment, and pharmaceuticals.^2−6^ Therefore, valorizing this waste fraction could improve the economic feasibility of fermentation processes while at the same time reducing the ecological footprint.

Under normal growth conditions, fungal cell wall biosynthesis is coupled with growth. However, cell wall integrity (CWI) is constantly monitored, and upon certain defects, the CWI signaling pathway is activated. CWI response leads to the strengthening of the cell wall via enhancing the biosynthesis of the cell wall components.^7^ CWI has been a major subject when studying fungi, and *Saccharomyces cerevisiae*(*S. cerevisiae*) has been used as the main model organism to reveal the mechanism of this highly conserved pathway.^8,10^ Cell wall stress can occur during normal growth conditions or rapid changes in the surrounding environment such as pH, temperature, and osmolarity.^11−13^ In *S. cerevisiae*, these stress signals are detected via a group of highly *O-*mannosylated cell-surface sensor-transducers at the plasma membrane. Upon activation of the receptors, nucleotide exchange factors (GEFs) are recruited to the plasma membrane, where they interact and catalyze the nucleotide exchange, turning the GDP-bound G protein Rho1 into the active GTP-bound form. Rho1 is considered the master regulator of the CWI signaling pathway as it conveys stress signals from different sensors to various actors involved in cell wall biosynthesis.^7,10^

Activated Rho1 triggers a mitogen-activated protein kinase (MAPK) cascade via the intermediary protein kinase C (Pkc1). The MAPK cascade activates two transcription factors Rlm1 and SBF (Swi4/Swi6), which regulate the expression of genes encoding cell wall proteins or genes related to cell wall biosynthesis.^7,9^

**Figure 1:**
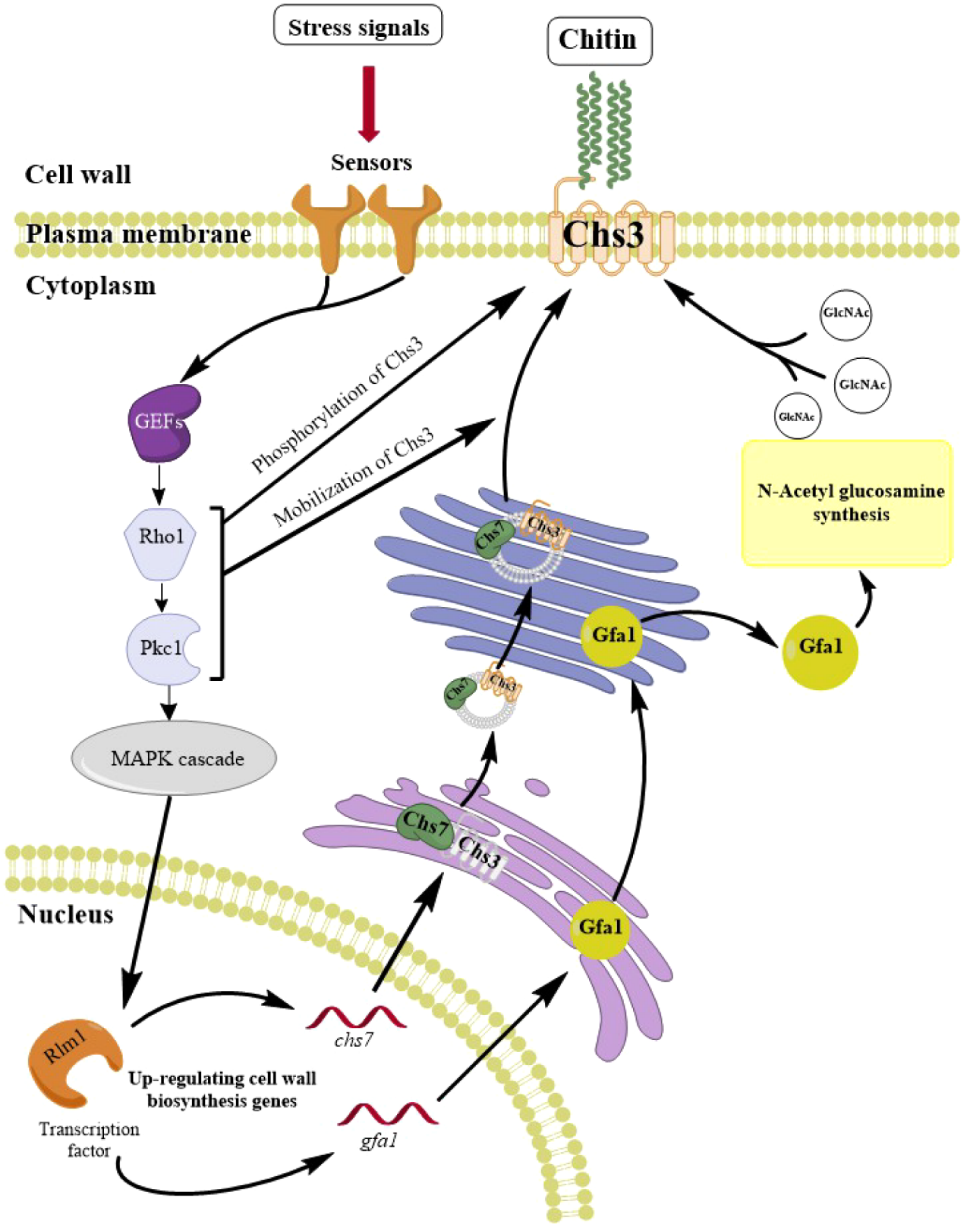
Cell wall integrity pathway (CWI) controls chitin synthesis in the cell wall of *S. cerevisiae*. The stress signals are detected by the sensors on the plasma membrane, which then recruit guanine exchange factors (GEFs) to activate the G-protein (Rho1). Subsequently, Rho1 interacts with and activates protein kinase C (Pkc1). The signal is then amplified through a MAPK cascade. One significant outcome of this pathway is the overexpression of transcription factor Rlm1, which regulates numerous genes related to cell wall biosynthesis. Rlm1 enhances chitin synthesis via the upregulation of *GFA1* to increase the production of chitin precursor GlcNAc, and *CHS7* to aid the post-translational mobilization of the primary chitin synthesis enzyme in yeast, Chs3. Besides promoting chitin synthesis via the MAPK cascade, Rho1 and Pkc1 also play essential roles in the phosphorylation and mobilization of Chs3 to the plasma membrane.

One of the primary outcomes of the CWI response is enhancing chitin synthesis to strengthen the cell wall, a process that is mediated via at least two mechanisms (Figure 1).^10,14,15^ First, Pkc1 and Rho1 mobilize chitin synthase 3 (Chs3), the main chitin synthesis enzyme, from the chitosome to the plasma membrane. This process occurs immediately and independently from the effectors of the MAPK cascade.^16^ Second, the production of the chitin precursor GlcNAc increases via the induction of the glucosamine-6-phosphate synthase gene (*GFA1*) by the Rlm1 transcription factor.^15,17^ There is a third, more controversial, mechanism postulating that Rlm1 upregulates *chs3* expression.^7,14,18^ However, there are contradicting studies reporting neither an increase in *CHS3* mRNA nor protein in *Δgas1* and *Δfks1* yeasts, respectively.^19,20^ Instead, the deletion of *fks1* led to a significant increase in the expression of *CHS7*, which plays a vital role in the mobilization of Chs3.^19,21^ Moreover, simultaneously, activated Rho1 also directly interacts with and regulates proteins involved in glucan synthesis, namely β-1,3-glucan synthase, β-1,6-glucan synthase, actin organization (Bni1, Bnr1), and exocytosis (Sec3).^7^

In this study, we established and evaluated a new concept for coproduction in fungi using*S. cerevisiae*as a model organism. To this end, a genetic switch was developed that is based on the controlled expression of key regulators of CWI response – wild-type *RHO1* and *PKC1* and their constitutively active mutant forms. The genetic switch was evaluated in two typical biotechnological production scenarios: one involving the coproduction of an intracellular, endogenous product (storage lipids) and chitin, while the other involving the coproduction of an overexpressed secreted protein (acid phosphatase) and chitin. Moreover, given the high conservation of the CWI response across fungal species, our technology holds significant promise for integration into chitin-rich fungi, potentially advancing the efficiency of fungal chitin production.

## 2 Results and Discussion

Here, we developed an approach that enables valorizing the residual biomass of yeast cells by creating revenues from the cell wall fraction. We devised an approach to target and increase the chitin content of the cell wall in a controlled manner. For this, we harnessed the constitutively active mutant forms of Rho1 (*RHO1*^*Q68H*^) or Pkc1 (*PKC1*^*R398A*^) that function in the absence of any cellular stress.^16,22,23^ Mutation changing a glutamine (Q) to a histidine (H) at position 68 traps Rho1 in its GTP-binding form, which in turn activates the protein constitutively.^24^ In the case of Pkc1, mutation changing an arginine (R) to an alanine (A) at position 398 makes the catalytic domain unable to bind to its own pseudosubstrate region, freezing Pkc1 in its constitutively activated form.^25^ Using wild-type and mutant forms of these regulatory proteins, we created a genetic toolbox to increase the chitin content and evaluated the setup in two different scenarios for the coproduction of biotechnologically valuable products.

### 2.1 Constitutive Overexpression of *RHO1* and *PKC1* and Its Mutant Forms Can Increase Chitin Content

**Figure 2:**
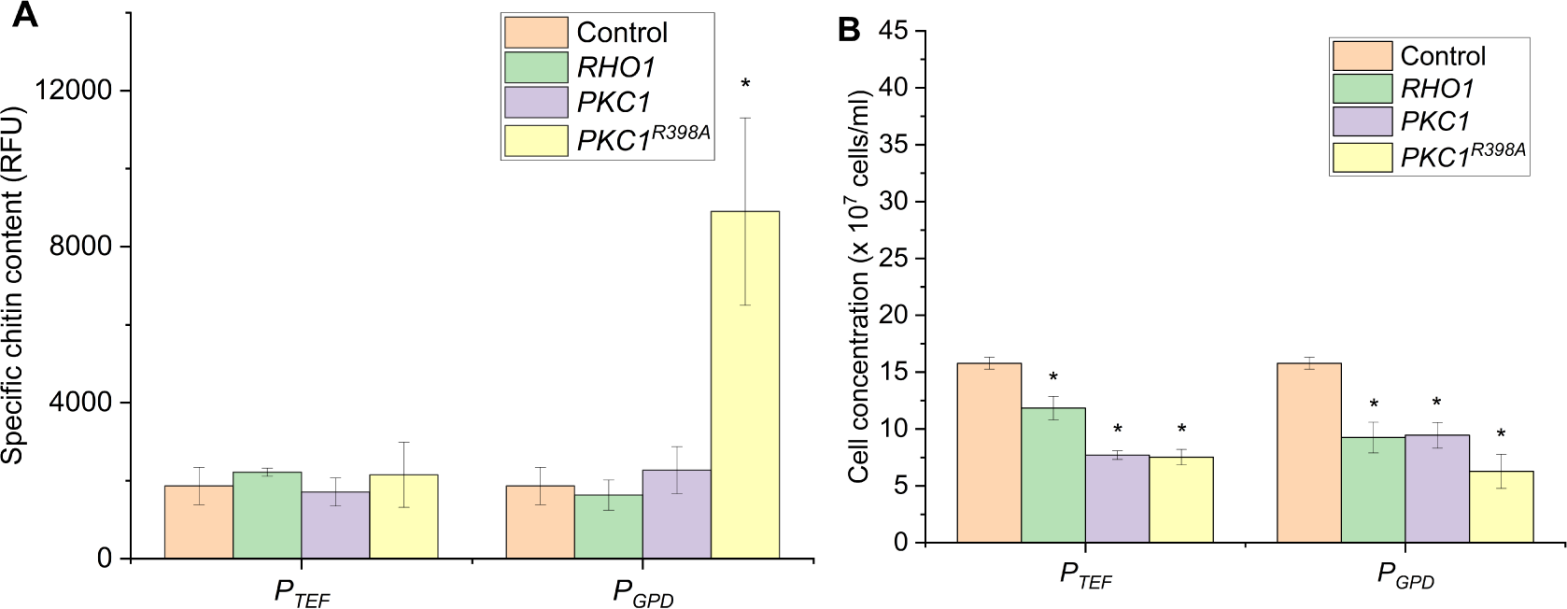
Specific chitin content (A) and final cell concentration (B) of yeast strains with overexpressed CWI genes under the control of constitutive promoters *P*_*TEF1*_ and *P*_*GPD*_. The control strain contained an empty plasmid. Cells were grown in SD-Ura containing 2% glucose for 24 h before chitin content, and the final cell concentrations were recorded. The specific chitin content is reported as relative fluorescence units (RFU) measured from staining 2 × 10^7^ cells with Calcofluor White. The data represent the mean and standard deviation from three independent experiments. *p < 0.05 indicates a significant difference compared to the control strain using a two-tailed Student’s *t* test.

First, we explored whether the regulatory genes related to the CWI pathway (including *RHO1*, *PKC1*, *RHO1*^*Q68H*^, and *PKC1*^*R398A*^) could be overexpressed under two commonly used constitutive promoters of different strengths, *P*_*TEF1*_ and *P*_*GPD*_, to determine their effects on the chitin content of *BY4742* yeast cells (Figure 2A). As anticipated, the overexpression of wild-type *RHO1* and *PKC1* had no significant effects on the chitin content of the yeast neither under *P*_*GPD*_ nor *P*_*TEF1*_. However, the chitin content increased significantly to about five times compared to the control when *PKC1*^*R398A*^ was overexpressed under the *GPD* promoter. Interestingly, the same gene overexpressed under *P*_*TEF1*_ did not result in a similar increase as *P*_*GPD*_. This could be due to the strength of the promoters, in which *P*_*GPD*_ is often considered stronger than *P*_*TEF1*_.^26,27^

Together with the chitin content, the final cell densities of the cultures were monitored. Generally, a negative effect on the growth was observed, with the magnitude of the reduction depending on the combination of the gene and the promoter (Figure 2B). However, the growth defects did not correlate with the corresponding increases in chitin content. The most severe effect was experienced in yeast strains overexpressing *RHO1*^*Q68H*^, where no cell growth could be seen in any liquid culture. The toxicity of constitutive activation of the CWI on yeast growth was documented in previous studies.^23,28^

While these findings support the concept that the chitin content can be increased by genetic means, they also indicate that a more sophisticated approach with a more fine-tuned expression of these regulatory proteins would be required to lower the negative impact on growth.

### 2.2 Inducible Promoters Enable to Optimize Chitin Production While Minimizing Growth Defects

We selected two different types of promoters: the copper-inducible *CUP1* promoter (*P*_*CUP1*_) and the galactose-inducible *GAL1* promoter (*P*_*GAL1*_). *P*_*CUP1*_ enables induction in the presence of different carbon sources, and its application is therefore more versatile. In contrast, *P*_*GAL1*_ enables expression in the presence of galactose, which at the same time can also serve as a carbon source.

**Figure 3:**
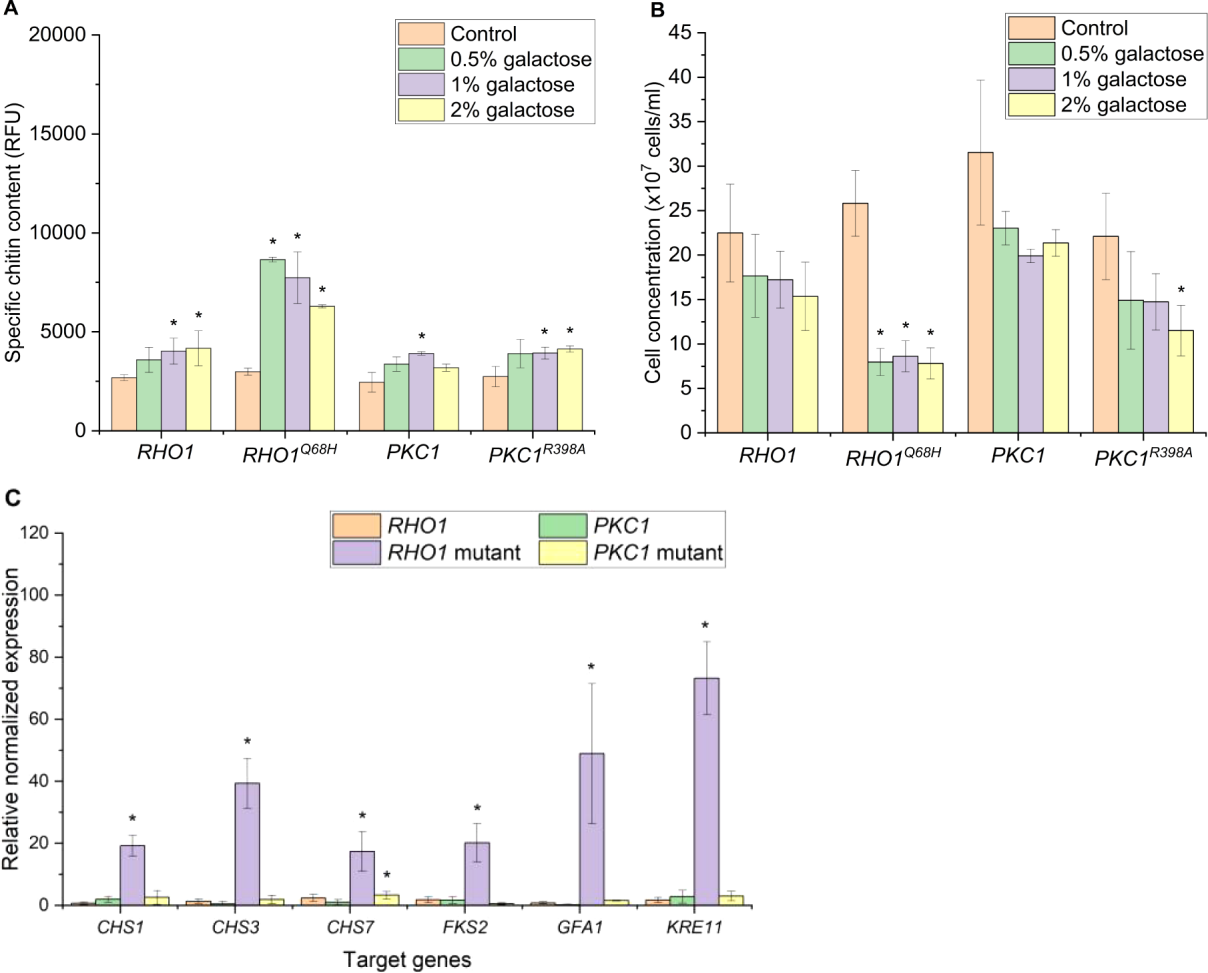
Specific chitin content (A) and final cell concentration (B) of yeast strains with overexpressed CWI genes under the control of the *GAL1* promoter (*P*_*GAL1*_). Samples were analyzed 18 h after induction with various galactose concentrations (0% to 2%). Noninduced cultures served as controls. The specific chitin content is reported as relative fluorescence units (RFU) measured from staining 2 × 10^7^ cells with Calcofluor White. The data represent the mean and standard deviation from three independent experiments. (C) Expression of CWI target genes analyzed by real-time PCR. RNAs were extracted from yeast samples 18 h after induction with 0.5% galactose, and noninduced cultures served as controls. The data represent the mean and standard deviation from a minimal of two independent experiments. *p < 0.05 indicates a significant difference compared to the control strain using unpaired Student’s *t* test.

We first tested the galactose-inducible promoter for expression of the regulatory genes by using a range of galactose concentrations for induction. Notably, the yeast strain overexpressing *RHO1*^*Q68H*^ under *P*_*GAL1*_ was able to grow at all tested inducer concentrations, showing that shifting to an inducible promoter was helpful. The highest chitin content (8656.33 RFU), which was about three times higher than the noninduced control, was recorded with the yeast overexpressing the *RHO1*^*Q68H*^ gene when induced with 0.5% galactose. Thus, the chitin content was similar to the level observed in the yeast strain overexpressing *PKC1*^*R398A*^ under the control of the *P*_*GPD*_. Interestingly, increasing the galactose concentration to 1% and 2% did not result in a further increase in the chitin content in the yeast cell wall (Figure 3A). In contrast, overexpression of *PKC1*^*R398*^ and the wild-type forms, *RHO1* and *PKC1,* only slightly increased the chitin content of the yeasts, with improvements of 1.3 to 1.5 times compared to the noninduced control across all tested galactose concentrations.

Despite the ability of *P*_*GAL1*_*-RHO1*^*Q68H*^ to grow using this experimental setup, the negative impact of its expression on the growth remained substantial. We observed an approximately 70% reduction in the final cell concentrations compared to the noninduced strain across all tested galactose concentrations. Despite the modest increase in chitin, strains expressing *RHO1*, *PKC1*, and *PKC1*^*R398A*^ exhibited noticeable, yet mostly statistically nonsignificant, growth defects. Notably, when induced with 2% galactose, the *PKC1*^*R398A*^ strain showed a 50% reduction in the final cell concentration. The negative effects on growth were milder in the strains with *P*_*GAL1*_*-RHO1* and *P*_*GAL1*_*-PKC1*, with reduction in the final cell concentrations ranging from roughly 20% to 30% compared to the noninduced control. Similar effects were observed when these genes were overexpressed under constitutive promoters (Figure 3B).

Moreover, we analyzed whether the activation of the regulatory genes leads to the induction of genes that are under the control of the CWI response. For induction, we used the galactose concentration that led to the highest chitin production. We selected genes that are directly or indirectly involved in chitin synthesis namely *CHS1*, *CHS3*, and *CHS7*. Furthermore, *GFA1*, a gene required for the synthesis of the chitin precursor molecule GlcNAc was included. In addition, we selected two genes that are involved in beta-glucan synthesis, *KRE11* and *FKS2*. As expected, all selected genes were significantly upregulated when *RHO1*^*Q68H*^ was overexpressed, with the highest increases seen in the expression of *CHS1, GFA1,* and *KRE11*, reaching more than 40-fold higher expression levels compared to the noninduced control (Figure 3C). Meanwhile, overexpressing *RHO1, PKC1,* and *PKC1*^*R398A*^ with 0.5% galactose led to no significant increase among all the targeted genes, except for a 4-fold increase in the expression of *CHS7* in *P*_*GAL1*_*-PKC1*^*R398A*^ strain. The expression data correspond well with the increases in specific chitin content, where the highest increase in chitin content was witnessed in *RHO1*^*Q68H*^ overexpressing yeast. A previous study also showed that *CHS7* overexpression alone did not lead to changes in the chitin content in yeast, but the combined upregulation of *GFA1*, *CHS3*, and *CHS7* could increase chitin accumulation in yeast up to 2.9-fold.^14^

Overall, using the inducible *P*_*GAL1*_, the expression of CWI genes allowed us to successfully increase the chitin content using both regulatory proteins. The most significant increase was recorded in yeast overexpressing *RHO1*^*Q68H*^, nearly matching the level achieved with the constitutive promoters. At the same time, attenuated growth defects were observed, as most clearly evidenced by the growth of the yeast strain overexpressing the mutant *RHO1*^*Q68H*^ gene. As expected, the activation of *RHO1*^*Q68H*^ led to the induction of genes related to cell wall biosynthesis, including chitin synthesis-related genes.

**Figure 4:**
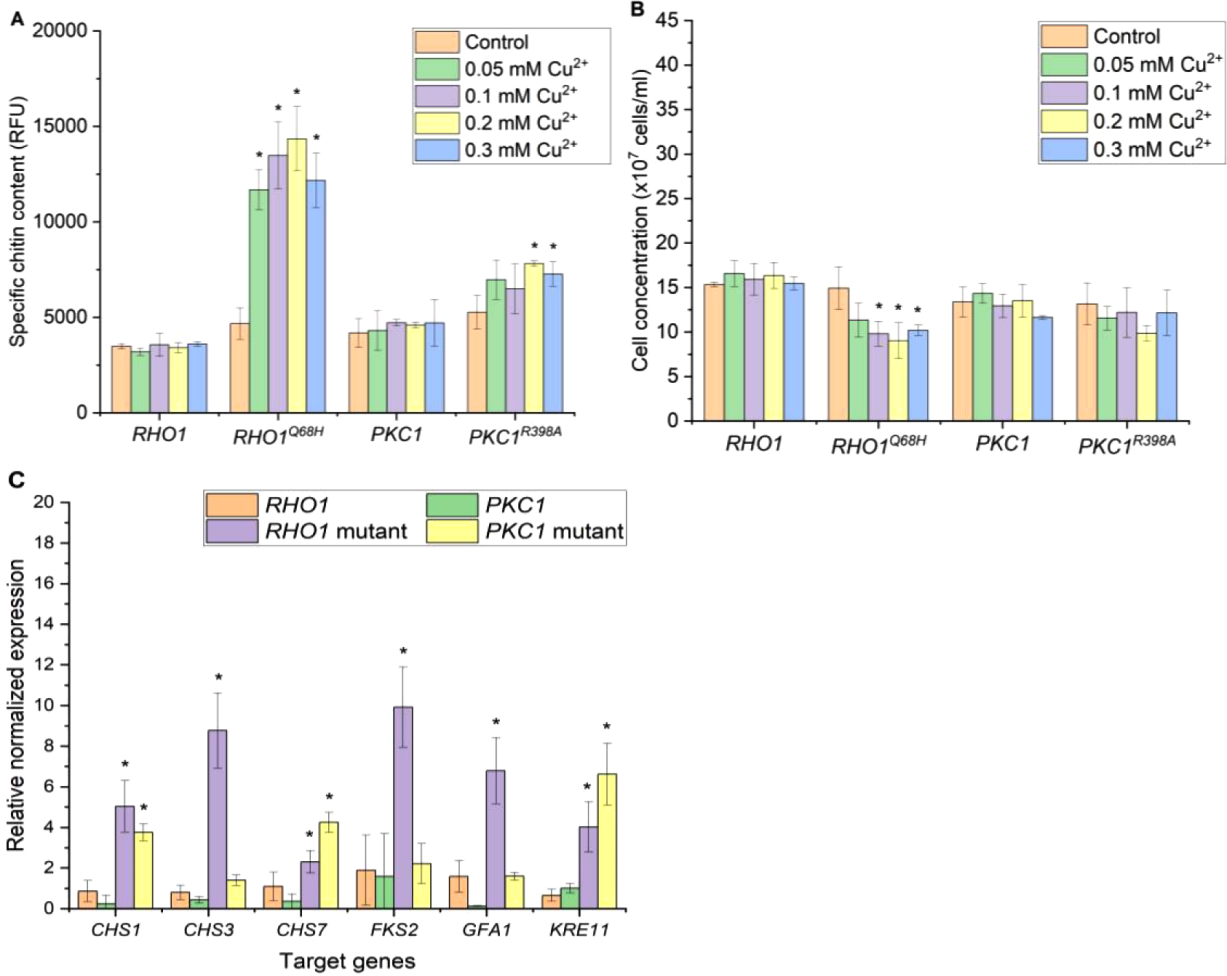
Specific chitin content (A) and final cell concentration (B) of the yeast strain with overexpressed CWI genes under the *CUP1* promoter (*P*_*CUP1*_). Samples were analyzed 18 h after induction with various Cu^2+^ concentrations (0 to 0.3 mM). Noninduced cultures served as controls. The specific chitin content is reported as relative fluorescence units (RFU) measured from staining 2 × 10^7^ cells with Calcofluor White. The data represent the mean and standard deviation from three independent experiments. (C) Expression of CWI target genes analyzed by real-time PCR. RNAs were extracted from yeast samples 18 h after induction with 0.2 mM CuSO_4_, and noninduced cultures served as controls. The data represent the mean and standard deviation from three independent experiments. *p < 0.05 indicates a significant difference compared to the control strain using unpaired Student’s *t* test.

To increase the versatility of the switch, we also tested overexpressing the target genes under *P*_*CUP1*_, since the expression from *P*_*CUP1*_ does not restrict the use of certain carbon sources. Similar to the results with *P*_*GAL1*_, the yeast strain overexpressing *RHO1*^*Q68H*^ was also viable and exhibited the most significant increase in chitin content (Figure 4A). Notably, the highest chitin reading surpassed 14000 RFU when 0.2 mM Cu^2+^ was introduced, marking the highest chitin level recorded across all promoters tested so far. Furthermore, the chitin content of the *P*_*CUP1*_*-PKC1*^*R398A*^ yeast strain also reached nearly 8000 RFU when induced with 0.2 mM Cu^2+^, nearly doubling the highest recorded chitin content for this gene when expressed from *P*_*GAL1*_. The chitin content of *P*_*CUP1*_*-RHO1* and *P*_*CUP1*_*-PKC1* when induced with Cu^2+^ did not increase significantly, resembling what was observed when using *P*_*GAL1*_. While the use of *P*_*CUP1*_ proved to be beneficial, basal activation of the promoter was taking place due to the presence of Cu^2+^ in the medium.

Furthermore, the negative effects on the growth of the yeast overexpressing these genes were significantly reduced with the *P*_*CUP1*_ setup compared to the *P*_*GAL1*_ setup (Figure 4B). There were no significant reductions in the final cell concentration of *P*_*CUP1*_*-PKC1, RHO1,* and *PKC1*^*R398A*^ yeast strains across all tested Cu^2+^ concentrations compared to the noninduced control cultures. Moreover, we observed a reduction of only 40% in the final cell concentration of the best-performing yeast strain, *P*_*CUP1*_*-RHO1*^*Q68H*^, induced with 0.2 mM CuS0_4_.

We analyzed the expression of the same set of genes as described above. For the gene expression analysis, we induced the strains with 0.2 mM Cu^2+^ that resulted in the highest chitin content. Overexpressing *RHO1*^*Q68H*^ under *P*_*CUP1*_ led to significant increases in the expression of all selected genes but with a smaller magnitude compared to that in the *P*_*GAL1*_ experiment (Figure 4C). However, it is worth noting that *P*_*CUP1*_ is leaky due to the presence of Cu^2+^ in the medium. Therefore, the noninduced control in the *P*_*CUP1*_ experiment may have already been slightly stimulated by the expression of *RHO1*^*Q68H*^, which could affect the basal expression of the selected set of genes. Furthermore, similar to the experiment with the *GAL1* promoter, neither *RHO1* nor *PKC1* overexpression under *P*_*CUP1*_ led to significant increases in the expression of tested genes. Interestingly, *PKC1*^*R398A*^ overexpression significantly increased the expressions of *KRE11, CHS7, and CHS1* to levels comparable to or even higher than those observed with *RHO1*^*Q68H*^ overexpression. However, the specific chitin content of the *P*_*CUP1*_*-RHO1*^*Q68H*^ strain is nearly double that the value of the *P*_*CUP1*_*-PKC1*^*R398A*^ strain. This suggests that in addition to *CHS1* and *CHS7*, the upregulation of *CHS3* and *GFA1* is essential for high chitin accumulation in the yeast cell wall. This finding is in agreement with a study from Lagorce et al. (2002), which showed that yeast strains overexpressing *GFA1*, *CHS3,* and *CHS7* exhibited the highest chitin content compared to strains overexpressing only one or two of these genes.^14^

In summary, using an inducible promoter to overexpress the target genes led to significant improvements in terms of chitin content as well as cell growth compared to constitutive promoters. Furthermore, gene expression data indicate that the overexpression of *RHO1*^*Q68H*^ in both inducible promoter setups successfully upregulated genes related to chitin synthesis (*CHS1*, *CHS3*, *CHS7*, and *GFA1*). Thus, *RHO1*^*Q68H*^ proved to be the most promising candidate to enhance the chitin content.

### 2.3 Genetic Switch Functions Irrespective of the Growth Phase

Our previous results suggest that overexpressing *RHO1*^*Q68H*^ leads to a significant increase in chitin content in *BY4742* yeast cells with both *P*_*GAL1*_ and *P*_*CUP1*_ when induced at the lag or early exponential phase (early induction). However, during manufacturing at the industrial scale, often fermentation schemes are used where the biomass production phase is separated from the production phase. Therefore, we explored an alternative induction strategy, evaluating induction at late exponential or early stationary growth phases (late induction). The study by Etcheverry in 1990 demonstrated that *P*_*CUP1*_ is suitable for this approach by successfully expressing the human serum albumin gene in *S. cerevisiae* at an OD_600_ of around 10. Therefore, the *P*_*CUP1*_ setup was evaluated in this experiment.

**Figure 5:**
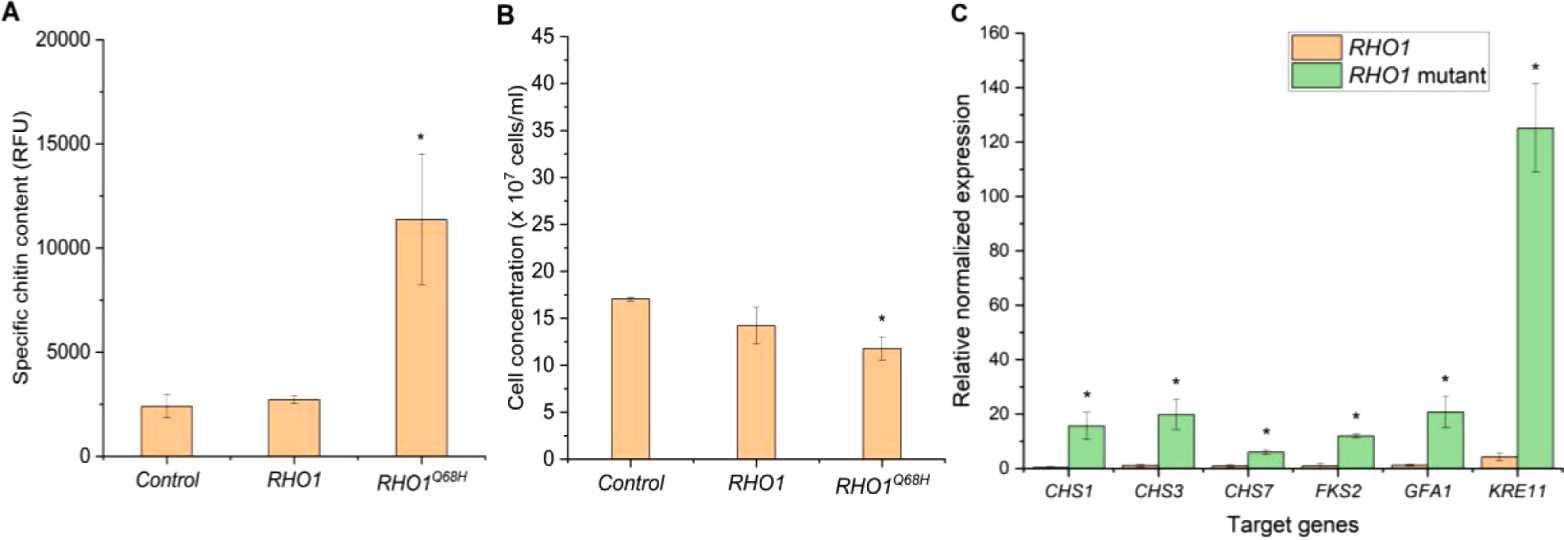
Specific chitin content (A) and cell concentration (B) of the late induction sample. The specific chitin content is reported as relative fluorescence units (RFU) measured from staining 2 × 10^7^ cells with Calcofluor White. The data represent the mean and standard deviation from three independent experiments. (C) Expression of CWI target genes analyzed by real-time PCR. RNAs were extracted from yeast samples 24 h after induction with 0.2 mM CuSO_4_, and noninduced empty plasmid cultures served as controls. The data represent the mean and standard deviation from three independent experiments. *p < 0.05 indicates a significant difference compared to the control strain using unpaired Student’s *t* test.

We included only *RHO1*^*Q68H*^ and two controls, the wild-type control and the empty plasmid control. Therefore, we modified the medium to include sodium phosphate buffer (data not shown). Under the optimized conditions, the chitin content of the cells overexpressing *RHO1*^*Q68H*^ reached above 11000 RFU, which is slightly lower than in that in the early induction experiment (Figure 5A). However, the final cell concentration of this strain was more than 11 × 10^7^ cells/ml, which is an improvement compared to the early induction (around 9 × 10^7^ cells/ml) (Figure 5B). To compare the chitin yield from early and late induction experiments, we calculated the volumetric chitin content. The volumetric chitin content of cultivations with the late induction scheme was about 67700 RFU/ml and was higher than the content from cultures with early induction (about 63800 RFU/ml). However, a two-tailed Student’s *t*-test indicates that the differences in volumetric chitin content were not significant (*p* = 0.802). Additionally, we also evaluate the expression of cell wall biosynthesis-related genes at the end of the cultivation process by performing qPCR with the same set of genes chosen as earlier. As expected, the yeast strain with high chitin accumulation (*RHO1*^*Q68H*^ overexpressed) shows the upregulation of all selected genes (Figure 5C). We observed a change in the gene expression pattern compared to the early induction experiment; however, the time points for induction and for sampling RNA were also changed, thus, limiting comparability of the two sets of experiments. However, we have demonstrated that our genetic switch is equally effective at enhancing the chitin content at both the early and late cultivation phases.

### 2.4 Chitin Accumulates in the Whole Cell Wall of Yeast Cells

So far, we have successfully developed a flexible genetic switch that can increase the chitin content of the yeast cell wall by up to 5 times at either early or late cultivation phases using *P*_*CUP1*_ controlling *RHO1*^*Q68H*^ expression.

In vegetative yeast cells, chitin accumulates around the septum.^15,29,30^ However, as the chitin content in the cell wall increases in response to our induction systems, we explored the sites of these additional chitin deposits in the cell wall using fluorescence microscopy of Calcofluor White-stained cells. For this analysis, we chose cells that were expressing *RHO1*, *RHO1*^*Q68H*^, and a control and were cultivated with the so far most powerful setup for chitin production, *P*_*CUP1*_.

**Figure 6:**
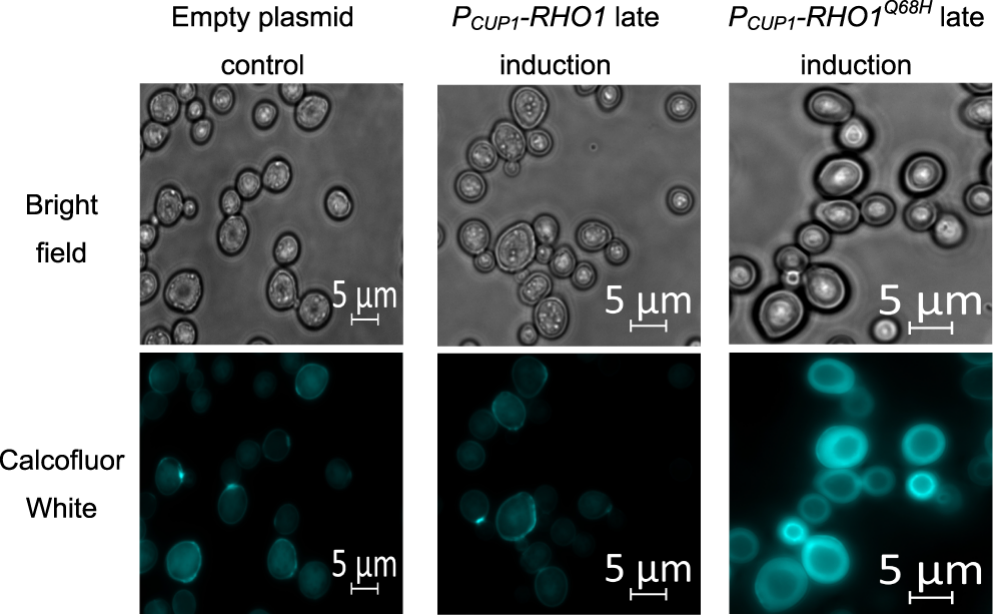
Fluorescence microscopy images of yeast cells stained with Calcofluor White. Chitin distribution in the cell wall is compared between a wild-type yeast containing an empty plasmid and yeast that are overexpressing *RHO1* and *RHO1*^*Q68H*^ under *P*_*CUP1*_. The analyzed yeasts were cultivated following the late induction setup of *P*_*CUP1*_.

In the control samples, very localized staining was visible. These stained structures are found in cells that are budding and are identical to the septum (Figure 6). Similar stained structures were also visible in cells expressing *RHO1*. In contrast, a strong fluorescence signal was emitted from the periphery of the cell in yeast cells expressing *RHO1*^*Q68H*^. This indicates that in yeast with induced chitin synthesis, the whole cell wall is reinforced with chitin. Similar observations were made when analyzing *FKS1* and wild-type yeasts stained with Calcofluor White.^31^

### 2.5 Coproduction of Chitin and Storage Lipids in Yeast Cells

Exploiting coproduced chitin as a side stream is recognized as a promising, sustainable, and cost-effective approach for naturally chitin-rich yeasts and fungi. For example, studies have demonstrated the potential of coproducing chitin and lactic acid, as well as chitin and fumaric acid, using *Rhizopus oryzae* or chitin as a side stream from the fermentation of *Aspergillus niger* for citric acid production.^32−34^ Therefore, we addressed whether the previously developed approach to genetically increase chitin content can be integrated with the production of two model products: storage lipids that accumulate intracellularly and an overexpressed secreted enzyme, acid phosphatase. Furthermore, as the CWI response is conserved among fungal species, successful coproduction in our model system would indicate that chitin yield could be improved in coproduction setups with chitin-rich fungi by actively controlling the CWI response.

**Figure 7:**
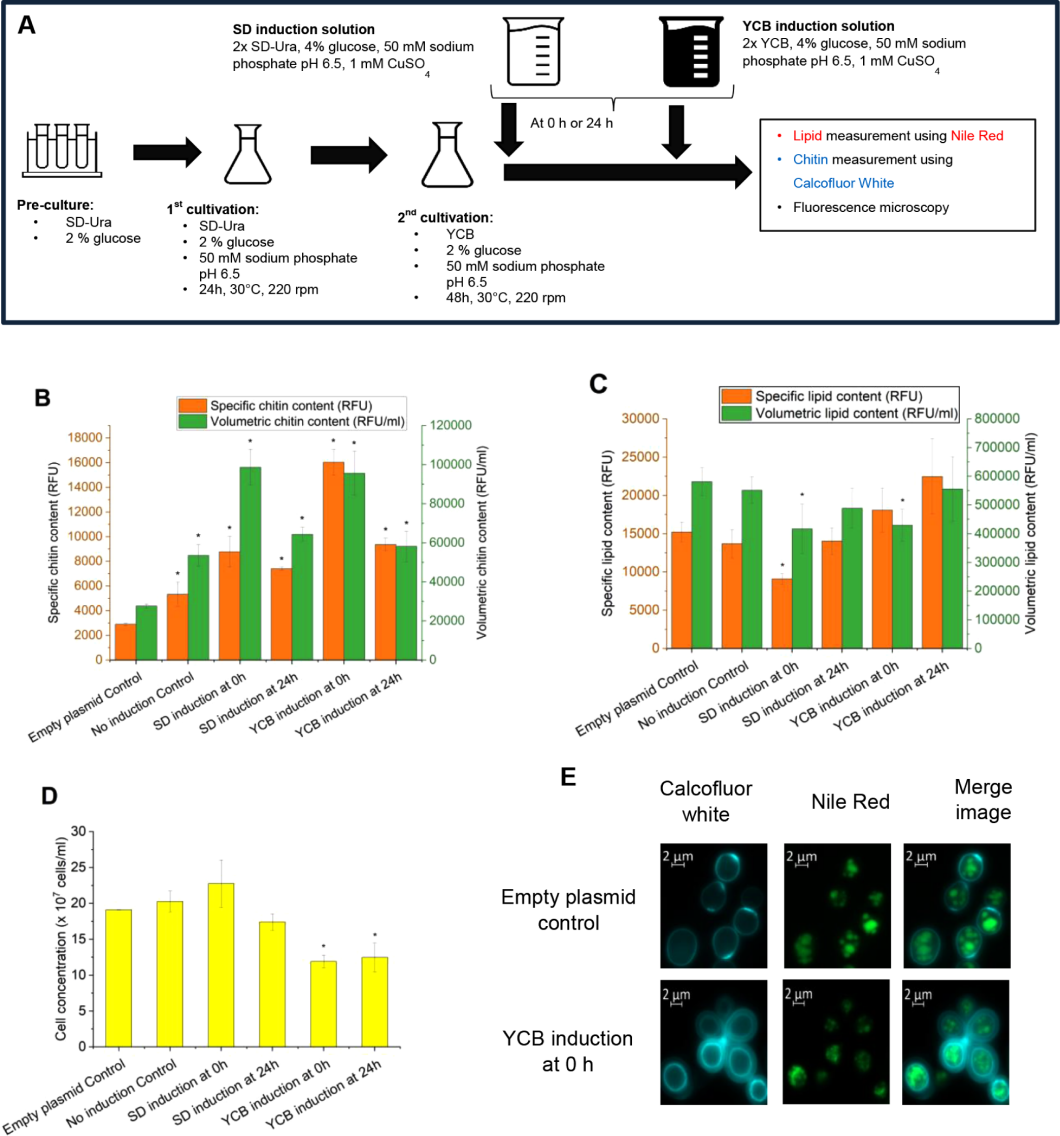
Lipid and chitin coproduction using YAN45 and YAN46 (control) strains. (A) Cultivation scheme to coproduce chitin and lipid in yeast using different induction strategies; (B) specific and volumetric chitin content of yeasts. The specific chitin content is reported as relative fluorescence units (RFU) measured from staining 2 × 10^7^ cells with Calcofluor White, while the volumetric chitin content is reported as relative fluorescence units of the total amount of cells per milliliter (RFU/ml); (C) specific and volumetric lipid content of yeasts. The specific lipid content is reported as relative fluorescence units (RFU) measured from staining 5 × 10^6^ cells with Nile Red, while the volumetric lipid content is reported as relative fluorescence units of the total amount of cells per milliliter (RFU/ml); (D) cell concentrations were measured using a hemacytometer; (E) fluorescence microscope images of yeast strains stained with Calcofluor White and Nile Red. The data in panels B to D represent the mean and standard deviation from three independent experiments. *p < 0.05 indicates a significant difference compared to the control strain using unpaired Student’s *t* test.

To coproduce lipid and chitin in yeast, the *P*_*CUP1*_*-RHO1*^*Q68H*^ switch was transferred into a yeast strain devoid of the two main lipases Tlg3p and Tlg4p responsible for storage lipid mobilization. The strains were grown according to the scheme shown in Figure 7A, which included a phase to expand biomass, followed by lipid accumulation for 48 h. Two approaches were followed for induction: one providing the inducer together with additional nitrogen-limited, lipid production medium (YCB), while the other was based on the idea to provide limited amounts of nitrogen as GlcNAc biosynthesis requires nitrogen and was administered with an SD medium-based solution. The switch for chitin production was activated either immediately after changing the medium (0 h) or 24 h later. We included two controls: one that lacked the switch, while the other contained the switch but was not induced. However, as the medium used for cultivation contains CuSO_4_, a mild induction was taking place, leading to an increased chitin content with no negative effects on lipid accumulation or growth, as shown in Figure 7B–D.

All induction regimes led to higher specific chitin content compared to that of the control strains. Induction right after starting lipid accumulation led to a higher specific chitin content compared to induction after 24 h. This difference is less prominent when using the SD-based induction (around 1300 RFU difference) but is very substantial when using YCB-based induction (around 7000 RFU difference). Additionally, the early induction approach in the latter treatment recorded the highest chitin signal so far, above 16000 RFU; nearly five times higher chitin content was observed with the empty plasmid control (around 2900 RFU). Interestingly, the additional nitrogen introduced through the SD induction solution did not result in higher chitin production compared to YCB induction in any tested case; thus, chitin production was not limited by the YCB medium.

Compared to the control, only early induction with SD led to a statistically significant reduced level of lipid accumulation in the cells (around 40% reduction), while the other treatments did not lead to statistically significant differences (Figure 7C). This confirmed the possibility of increasing the chitin content of the yeast cells without affecting the lipid yield, the primary intracellular product. When comparing the different induction schemes, a trend toward higher specific lipid levels was seen in cultures induced with YCB-based induction solution compared to SD induction solution. The reduction in specific lipid content most likely originates from the availability of additional nitrogen in the culture medium, as nitrogen depletion increases lipid content in yeast and fungi.^35−37^

While we wanted to see the effects on specific productivity, we also report here the volumetric chitin and lipid contents, respectively, to better represent the overall productivity achieved in cultivations. When taking into account the final cell concentrations, the total volumetric chitin content of yeast cultures induced right after lipid accumulation increased significantly compared to that of the induction after 24 h (Figure 7B). However, unlike the specific chitin productivity, the differences in the volumetric chitin yield between the early and late approaches were equally prominent in either SD or YCB-based induction solution. Furthermore, despite the specific chitin content of the YCB-based induction at 0 h being the highest among all tested induction regimes and nearly 2 times higher than that of the SD-based induction, both treatments resulted in the same volumetric chitin content of more than 90000 RFU/ml which was roughly four times higher compared to yeast cells without the switch. This was because the negative effect on the growth was negligible when yeasts were induced with the SD induction solution, while nearly 40% reduction in the final cell concentrations compared to the empty plasmid control was seen in YCB-induced yeasts at both induction time points (Figure 7D).

As a result, when analyzing the volumetric chitin content, one can conclude that maximized volumetric chitin yield can be obtained in two ways: either with a modest increase in specific chitin content but an increase in total biomass (SD induction at 0 h) or by optimizing cellular chitin content but at the cost of reduced cell growth (YCB induction at 0 h) (Figure 7B,D). Additionally, when calculating the volumetric lipid yield, the early induction approaches led to about 20% reduction in the lipid accumulation compared to the controls, while late induction approaches did not (Figure 7C). Therefore, by adjusting the induction time, it is possible to amplify the chitin yields more than 2-fold without affecting the lipid yield (Figure 7B,C). Alternatively, a more significant increase in the chitin yield (up to four times) could also be achieved, but with a minor decrease in the volumetric lipid accumulation (roughly 20%).

Moreover, to confirm that lipid droplets and chitin accumulated in the same cells, we analyzed cells at the end of the cultivation by fluorescence microscopy with dual staining using Nile Red and Calcofluor White (Figure 7E). We included control cells harboring the empty plasmid and cells harboring the switch and induced with YCB at a 0 h sample. The Calcofluor White staining resulted in cells with thicker and brighter cyan fluorescence rings indicative of the cell wall in the YCB-induced sample compared to the empty plasmid control. This morphological change was also seen in the late induction experiment with *P_CUP1_* (Figure 6). Furthermore, the lipid droplets represented by green fluorescence dots inside the cells were seen in all of the tested samples, including those cells with thickened cell wall structures. This evidence clearly illustrates that cells do coproduce both lipid and chitin. These microscopy results also support the fluorescence measurements reporting the increased chitin content of the strains containing the genetic CWI switch while showing no significant changes in lipid content per cell.

### 2.6 Coproduction of Acid Phosphatase and Chitin

**Figure 8:**
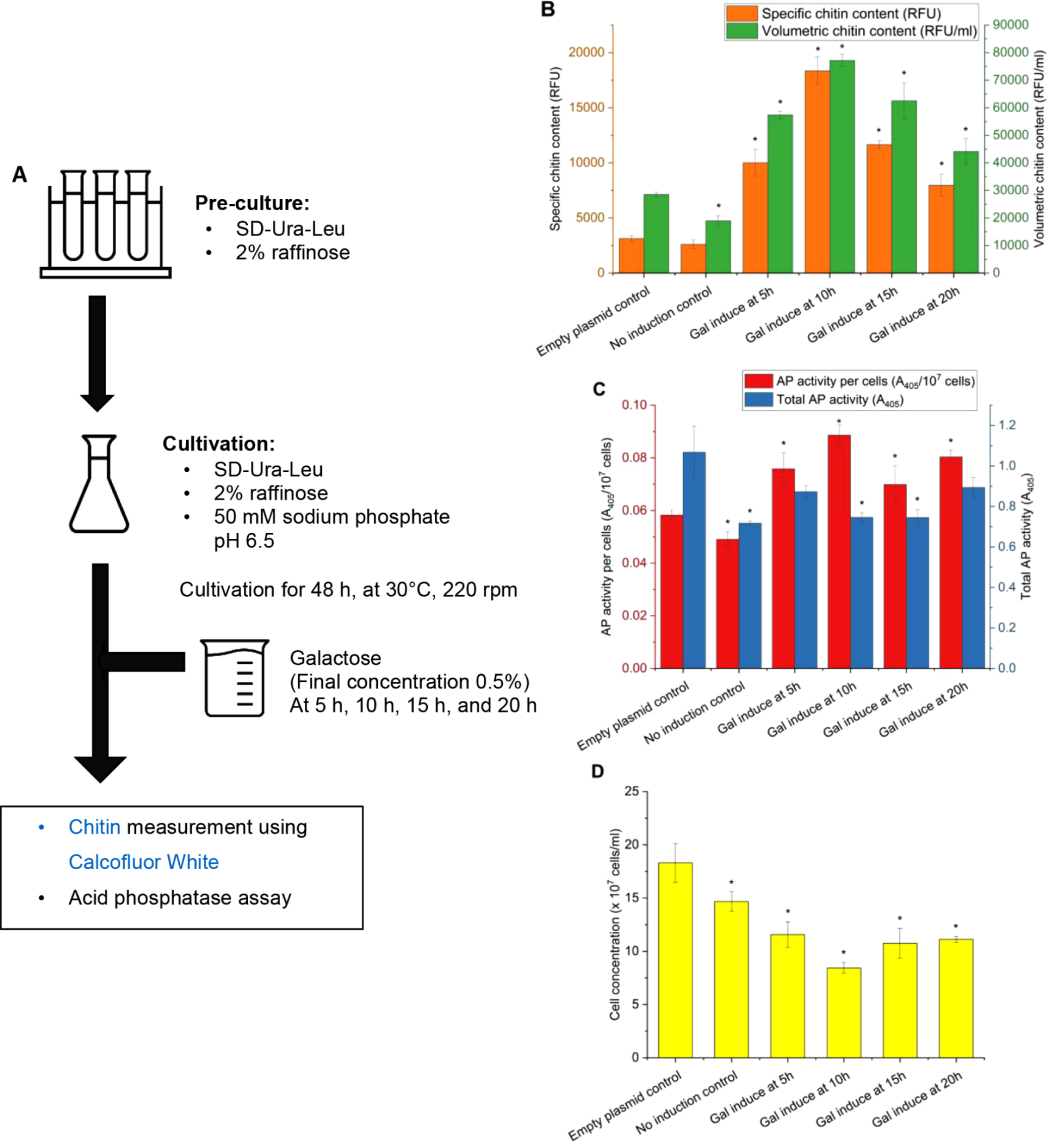
Acid phosphatase (AP) activity, chitin content, and cell concentration of AP and chitin coproducing yeast using YAN53 and YAN61 (control) strains. (A) Cultivation scheme to coproduce chitin and AP in yeast; (B) specific and volumetric chitin content of yeast strains induced with galactose at different time points. The specific chitin content is reported as relative fluorescence units (RFU) measured from staining 2 × 10^7^ cells with Calcofluor White, while the volumetric chitin content is reported as relative fluorescence units of the total amount of cells per milliliter (RFU/ml); (C) specific and total AP activity of yeasts induced with galactose at different time points. Total AP activity is represented as absorbance at 405 nm (*A*_405_), and the specific AP activity was calculated by dividing total AP activity by the cell concentration (*A*_405_/10^7^ cells); (D) cell concentrations were measured by using a hemacytometer. The data represent the mean and standard deviation from three independent experiments. *p < 0.05 indicates a significant difference compared to the control strain using unpaired Student’s *t* test.

Besides the intracellularly stored lipid, we also tested coproducing chitin with acid phosphatase, a secreted, extracellular product. We first introduced the construct for *P*_*CUP1*_*-RHO1*^*Q68H*^ into a yeast strain that constitutively expressed and secreted acid phosphatase into the culture medium. However, preliminary tests showed that Cu^2+^ ions completely inhibited acid phosphatase (data not shown). Therefore, we utilized the *P*_*GAL1*_*-RHO1*^*Q68H*^ switch to increase the chitin content in the AP-producing yeast, utilizing the lowest galactose concentration tested before, but varying the time point for induction as shown in Figure 8A. As in the previous experiment, we included two controls: one that contained an empty plasmid, while the other contained the switch but was not induced.

In summary, all induction regimes led to increased specific and volumetric chitin contents compared to the control strain. Optimal chitin production occurred when induction took place after 10 h of growth, reaching up to 18000 RFU in specific chitin content and up to 77000 RFU/ml in volumetric chitin content, approximately five times and 2.5 times higher, respectively, than those of the empty plasmid control. Although the increase in chitin content was less pronounced when induction took place earlier or later, they remained statistically significant (Figure 8B). Induction after 5 or 15 h resulted in roughly a 3-fold increase in specific chitin content and a 2-fold increase in the total chitin content compared to the empty plasmid control. The smallest rise in chitin content was observed in samples induced after 20 h, showing approximately a 2-fold increase in the specific chitin content and a 1.5-fold increase in volumetric chitin content. The difference in the magnitude of the increase in specific and volumetric chitin content was due to the negative effect on the growth of the yeast when *RHO1*^*Q68H*^ is overexpressed under *P*_*GAL1*_. As observed in other experiments with *P*_*GAL1*_, we observed a strong impact on growth, resulting in a nearly 40–50% reduction in the final cell concentrations across all induction regimes compared to the empty plasmid control, with the most severe effects seen in yeasts induced after 10 h (50% reduction) (Figure 8D).

Acid phosphatase activity per cell also increased compared to the control using all induction strategies with the highest activity seen in cultures when the switch was activated 10 h after inoculation (roughly 25% higher than the control sample). However, considering the negative effect on the growth when using *P*_*GAL1*_ setup, the total acid phosphatase activity measured in the culture medium of the sample with the highest chitin content only equaled roughly 75% of the acid phosphatase activity of the empty plasmid control. Interestingly, when galactose was added after 5 and 20 h, there were no statistically significant differences in the total secreted acid phosphatase activity between the induced cultures and the empty plasmid control (Figure 8C).

In summary, we demonstrated the potential of coproducing chitin with a secreted product (AP). By adjusting the induction time, producers can fine-tune the process to either maximize chitin yield while slightly compromising the total AP activity or to increase chitin yield to a lesser extent with no compromise on the total enzyme secreted.

### 2.7 Bioreactor Cultivations to Coproduce Chitin in Yeast Cells

Finally, we evaluated the scalability of the two coproduction systems in benchtop bioreactors. We chose the strain coproducing acid phosphatase and chitin and the strain coproducing storage lipids and chitin, respectively. Both strains were transformed with either the inducible *RHO1*^*Q68H*^ constructs or the control plasmid.

We used the same medium and general growth conditions for bioreactor cultivations as for the small-scale cultivations, except that the additional buffer substance was omitted from the medium, and pH was maintained at pH 5.0. For induction, the conditions that had resulted in the highest chitin content increases as identified in the small-scale cultivation were used. Cultivations were stopped after 48 h; chitin content and secreted AP or lipid content, respectively, were determined.

Overall, the amount of extracted crude chitin from both coproduction systems increased more than 2-fold in the yeast strains when overexpressing *RHO1*^*Q68H*^ compared to the control strains. Moreover, the results of this gravimetric analysis align well with the results obtained with the CW staining method; increases of the same order of magnitude were seen in the volumetric chitin content. Interestingly, the negative effect of *RHO1*^*Q68H*^ expression on the growth as observed in the small-scale experiments was not seen at the benchtop bioreactor scale (Tables 1 and 2). Furthermore, the total cell dry weight (CDW) determined at the end of the cultivation of the yeast strains overexpressing *RHO1*^*Q68H*^ was significantly higher than that of the control strains despite the similarity in the cell number. Since yeast cell walls can account for up to 30% of the biomass, the difference in the correlation of cell concentration and CDW here could be due to the increase in the cell wall materials when *RHO1*^*Q68H*^ is overexpressed.

**Table 1:** Yield of Acid Phosphatase and Chitin Co-Production Using Benchtop Bioreactors

Samples	Cell concentration (× 10^7^ cells/ml)	Cell dry weight (CDW) (g)	Chitin content per cells (RFU)	Volumetric Chitin content (10^6^ × RFU/ml)	Crude Chitin (g)	AP assay per cells (Δ*A*_405_/10^7^ cells)	Total AP activity (Δ*A*_405_)
YAN61 (control)	10.25 ± 0.07^a^	1.55 ± 0.01	5503 ± 717	57.8 ± 7.1	0.57 ± 0.03	0.088 ± 0.008	0.91 ± 0.08
YAN53	10.05 ± 1.20	2.88 ± 0.08	12602 ± 1770	128.7 ± 2.7	1.32 ± 0.02	0.073 ± 0.040	0.71 ± 0.32

a All data represent the mean and standard deviation from two independent experiments.

**Table 2:** Yield of Lipid and Chitin Co-Production Using Benchtop Bioreactors

Samples	Cell concentration (× 10^7^ cells/ml)	Cell dry weight (CDW) (g)	Chitin content per cells (RFU)	Volumetric Chitin content (10^6^ × RFU/ml)	Crude Chitin (g)	Lipid per biomass (g/g)	Total lipid content (g)
YAN46 (control)	5.21 ± 0.16^a^	1.41 ± 0.05	5926 ± 1429	38.5 ± 8.1	0.51 ± 0.05	0.36 ± 0.03	0.51 ± 0.02
YAN45	6.3 ± 0.89	1.89 ± 0.13	14022 ± 818	110.8 ± 22.0	1.15 ± 0.19	0.14 ± 0.002	0.27 ± 0.01

a All data represent the mean and standard deviation from two independent experiments.

Besides successfully replicating the increase in chitin content from the shake flask to the bioreactor scale, we also noticed some negative effects. When we scaled up lipid and chitin coproduction, the total lipid content of the *RHO1*^*Q68H*^ expressing strain decreased nearly half compared to the level of the control strain. This decrease was not as severe in the small-scale experiment, where only about a 20% reduction of the total lipid content was seen in the *RHO1*^*Q68H*^ expressing strain compared to the control. Despite changes in the total lipid content, the compositions of the storage lipids in *RHO1*^*Q68H*^ expressing and control strains in bioreactor-scale experiments were similar. In contrast, in the case of AP and chitin coproduction, the total AP activity of the *RHO1*^*Q68H*^ expressing strain at the bioreactor scale was reduced to a similar extent compared to the control as observed in small-scale cultivation. In both cases, a roughly 25% reduction was observed.

**Figure 9:**
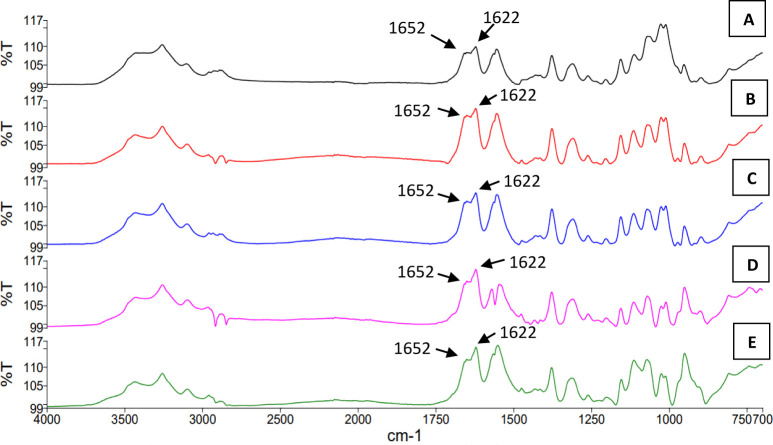
FTIR spectra of A. Commercially available chitin from crustacean, B. chitin extracted from the control strain YAN61 used for acid phosphatase and chitin coproduction experiment, C. chitin extracted from the *RHO1*^*Q68H*^ expressing strain YAN53 strain used for acid phosphatase and chitin coproduction experiment, D. chitin extracted from the control strain YAN46 used for lipid and chitin coproduction experiment, and E. chitin extracted from the *RHO1*^*Q68H*^ expressing strain YAN45 strain used for the lipid and chitin coproduction experiment.

Moreover, we evaluated whether the chemical footprint of the chitin isolated from the yeast cell wall is similar to the one obtained from crustaceans. Therefore, we compared the FT-IR spectrum of a commercial crustacean chitin with the spectra of chitin extracted from the cell wall of the four strains (Figure 9). The molecular footprints obtained by FT-IR revealed the signatures that are typical for α-chitin; the vibration modes of amide I show 2 peaks in the spectral region of 1660–1620 cm^–1^ (1652 and 1622 cm^–1^). Furthermore, the peaks observed for chitin extracted from yeasts in this study were also similar to the spectral signatures reported for chitin extracted from *Agaricus bisporus*.^38^ Thus, based on these molecular footprints, the chitin produced by the different yeast cells in this study was indistinguishable from crustacean chitin.

## 3 Conclusions

In conclusion, we successfully increased the chitin content in the yeast cell wall via the activation of the CWI pathway using various promoters and target genes. We identified *P*_*CUP1*_ and *RHO1*^*Q68H*^ as the most powerful combination for maximizing the chitin content, and the system can be induced early or late in the cultivation process. Expression data revealed that genes relevant for chitin synthesis were induced after the activation of *P*_*CUP1*_ and *RHO1*^*Q68H*^.

The data also confirmed the possibility of coproducing chitin in yeast cells that are producing other valuable products. We chose two distinct model products and two different cultivation schemes for coproduction. The first case inspected the possibility of combining the production of an intracellular endogenous product, lipid, with chitin. Our shake flask scale data show that chitin production can be optimized in such a way that lipid accumulation is not compromised. As the production of lipids and chitin is inducible, the results also indicated that there is a certain degree of freedom in how to perform the production process. The second case investigated the possibility of combining a recombinant protein with chitin production. Here, we utilized the constitutive expression of the secreted model enzyme and chitin. We observed a significantly reduced production of AP (25% reduction) but a strongly increased chitin accumulation in cells (five times increase). However, the induction time can also be tuned to reduce the negative effect on the total enzyme secreted while still significantly increasing the chitin yield.

Further enhancement of chitin accumulation in the cell wall could be attempted through medium optimization. Along these lines, Chagas et al. (2014) demonstrated that the chitin and glucan ratio in the yeast cell wall can be adjusted by cultivation conditions such as pH and temperature.^39^ Additionally, glucosamine addition in the cultivation medium was also proved to increase chitin content in the yeast cell wall.^15^

We further explored the scalability of our experiment by coproducing chitin with either lipids or AP in a benchtop bioreactor, using the same optimized conditions that previously yielded the highest specific chitin content at the shake flask scale. In both cases, chitin yield increased by approximately 2-fold. However, upon scaling up, we observed a more pronounced reduction in lipid accumulation (approximately 50%), while the decrease in the total AP activity remained consistent across both cultivation scales. Clearly, further work is required, including testing different induction strategies and medium optimization to minimize the effect on the yield of the primary product.

In general, our findings could help improve the sustainability and economic feasibility of manufacturing products via the fermentation of yeast cells. However, the economic feasibility will depend on the value of the primary product and putative reductions of its yield when coproducing chitin; here, a careful assessment would need to be performed. Furthermore, the strategies we demonstrated for actively controlling the CWI response to increase the chitin content in yeast are transferable to other naturally chitin-rich fungi. Given the conservation of the CWI pathway across fungal species, future work could investigate whether these approaches could be adapted to enhance chitin production in a variety of fungal systems. Exploring this possibility could unlock further industrial applications, extending beyond yeast to other fungal species, where chitin-rich biomass is of interest.

## 4 Materials and Methods

### 4.1 Plasmid Construction and Yeast Transformation

*RHO1* and *PKC1* were amplified from the genome of *S. cerevisiaeW303*α (*MAT*α *leu2–3,112 trp1–1 can1–100 ura3–1 ade2–1 his3–11,15*) using the KAPA2G hotstart Mastermix and the oligonucleotides listed in Table S1. The amplified genes were inserted either into pRS414 for subsequent mutagenesis or for expression into the shuttle vectors pRS416-Gal, pRS416-TEF, pRS416-GPD, and pJR025, a derivative of a pRS416 vector containing the CUP1 promoter (*P*_*CUP1*_). All plasmids used and created in this study are given in Table S2.

Point mutations were introduced into the *RHO1* and *PKC1* genes using the QuikChange Lightning Site-Directed Mutagenesis Kit (Aligent Tech) using either pRS414-*RHO1* or pRS414-*PKC1* as a template. The coding regions of the mutated genes *RHO1*^*Q68H*^ and *PKC1*^*R398A*^ were verified by sequencing. *RHO1*^*Q68H*^ and *PKC1*^*R398A*^ were then excised from the pRS414 backbone using *Sma*I/*Xho* I or *Sma*I/*Sal*I restriction enzymes, respectively, and ligated into the shuttle vectors for expression.

Plasmids were transformed into *S. cerevisiaeBY4742* (*MATα his3Δ1 leu2Δ0 lys2Δ0 ura3Δ0*) or a derivative thereof lacking *tlg3* and *tlg4* genes using the lithium acetate method.^40^ Solidified synthetic drop-out (SD) medium without uracil (SD-Ura) containing 0.67% w/v of yeast nitrogen base without amino acids (Sigma-Aldrich), 0.16% w/v CSM-Ura-His-Leu-Tryp drop-out mix, 2% w/v of glucose, 0.85% w/v histidine, 0.85% w/v leucine, 0.85% w/v tryptophan, and 2% w/v of agar was used for selection of transformants, except for the selection of strain YAN53, where SD agar without uracil and leucine (SD-Ura-Leu) was used. All strains used in this study are listed in Table S3.

### 4.2 Quantitative Real-Time-PCR

The total RNA extraction of yeast samples was done using the RNeasy Mini kit (Qiagen) and following the recommendations of the manufacturer. Extracted RNA samples were further treated with DNase to remove any DNA leftover using a TURBO DNA-free kit (Invitrogen). First-strand cDNAs were then synthesized from 200 to 300 ng of total RNA using a Maxima First Strand cDNA Synthesis Kit for RT-qPCR (Thermo Fisher Scientific). For each sample, a control for DNA contamination was made by performing the same reaction in the absence of the Maxima Enzyme mix. Real-time PCR was run with a CFXConnect thermocycler (Bio-Rad, USA), with each reaction containing 12.5 μL of Maxima SYBR Green qPCR Master Mix 2X (Thermo Fisher Scientific), 2 μL of the cDNA from the RT reaction, and 1.25 μL of one of the commercial PrimePCR SYBR Green Assay primers (Bio-Rad) specific for the target genes and ddH_2_O filled to a final volume of 25 μL. The identifiers for the primers are qSceCED0004953 (*CHS1*), qSceCED0003795 (*CHS3*), qSceCEP0013110 (*CHS7*), qSceCED0005280 (*GFA1*), qSceCED0002021 (*GAPDH*), qSceCED0002235 (*KRE11*), and qSceCED0002210 (*FKS2*). RT-PCR conditions were as follows: initial denaturation step at 95 °C for 10 min, 40 cycles at 95 °C for 15 s, 60 °C for 30 s, and 72 °C for 30 s. Additionally, a melting curve analysis was performed after the amplification to ensure the specificity of the reactions.

To quantify gene abundance, the expression level of each gene was quantified relative to the GADPH standard transcript, and the final relative gene expression data between the induced and noninduced samples were calculated using the 2^–ΔΔCt^ methods, as described by Livak and Schmittgen (2001).^41^ For late Cu^2+^ induction experiments, the expression data were normalized with the noninduced empty plasmid control culture. Results were analyzed with Bio-Rad CFX Manager 3.0 software.

### 4.3 Media and Culture Conditions

All precultures and experimental cultivations were conducted at 30 °C and 220 rpm unless stated otherwise. Experimental cultivations were performed in 24-well deep well plates (DWPs) with a 3 mL culture volume.

Precultures were grown in 3 mL of the SD-Ura or SD-Ura-Leu medium with 2% (w/v) glucose overnight, except for strains requiring induction with galactose, where glucose was substituted with 2% (w/v) raffinose.

On the next day, an appropriate volume of preculture was transferred to 3 mL of the selective medium to reach a starting OD_600_ of 0.2. For constitutive expression experiments, precultures were transferred to SD-Ura and cultivated for 24 h before harvesting samples. For experiments requiring galactose induction, the precultures were diluted in SD-Ura with 1% raffinose and incubated for 5 h before induction with different concentrations of galactose (0 to 2%). 1 mL of the sample was collected 24 h after dilution of cultures and stored at −20 °C.

For experiments with early induction with copper, various volumes of a 100 mM CuSO_4_ solution were added after 5 h of cultivation in SD-Ura to reach final Cu^2+^ concentrations ranging from 0 to 0.3 mM.

For experiments with late induction with copper, the precultures were diluted in the SD-Ura medium buffered with 50 mM sodium phosphate buffer pH 6.5. An SD induction solution (2 times concentrated SD-Ura, 50 mM sodium phosphate buffer pH 6.5, and 1 mM CuSO_4_) was added to the culture after 20 to 22 h of cultivation. The volume of the SD induction solution added was equivalent to one-fourth of the total cultivation volume. The cells were then grown for an additional 24 h before harvesting the samples.

For coproducing lipid and chitin, the required volumes of precultures were added to the SD-Ura medium supplemented with 50 mM sodium phosphate buffer at pH 6.5 and grown for 24 h. After that, the cells were collected by centrifugation at 3900 rpm for 5 min and washed twice with water. The cell pellets were resuspended in 3 mL of the Yeast Carbon Base without Uracil (YCB-Ura) medium for lipid production (0.67% w/v of yeast carbon base (Sigma-Aldrich), 0.85% w/v lysine, 0.85% w/v histidine, 0.85% w/v leucine, 0.85% w/v tryptophan, and 0.0002% w/v methionine, and 2% w/v of glucose) with 50 mM sodium phosphate buffer pH 6.5 and incubated for 48 h. Chitin production was induced by the addition of 0.75 mL of either the SD induction solution or YCB induction solution (2 times concentrated YCB-Ura, 50 mM sodium phosphate buffer pH 6.5, and 1 mM CuSO_4_). Cultures were induced either immediately after shifting the media (0 h of induction) or after 24 h of growth in YCB-Ura (24 h of induction). 2 mL of sample were collected 48 h after induction of cultures and stored at −20 °C.

For coproducing acid phosphatase (AP) and chitin, the required volumes of the precultures were added to the SD-Ura-Leu medium with 2% (w/v) raffinose and 50 mM sodium phosphate buffer at pH 6.5 and cultivated for 48 h. Induction was carried out with the addition of galactose to the final concentration of 0.5% (w/v) after 5, 10, 15, and 20 h of cultivation for chitin production. 2 mL samples of each culture were collected after 48 h of cultivation, from which one-half was frozen and used for chitin measurement, while the other half was immediately used for the AP assay.

### 4.4 Bioreactor Cultivations

All bioreactor cultivations for coproduction were conducted in 2 L Biostat Bplus bioreactors. The temperature of the bioreactor was set at 30 °C, and the pH was maintained at 5.0 by using 1N NaOH and 1N HCl, respectively. The dissolved oxygen level was kept above 30% by coupling the gas flow and stirring speed, ranging from 0 to 3 L/min and 200 to 800 rpm, respectively.

For the coproduction of acid phosphatase and chitin yeast strains, YAN53 and YAN61 were used. The first preculture was grown in 5 mL of the SD-Ura-Leu medium with 2% raffinose (w/v) for 2 days at 30 °C and 220 rpm before being transferred into 45 mL of the same medium to start the second preculture step in a 250 mL shake flask and incubated for 1 day under the same conditions. An appropriate volume of the second preculture was then added to the bioreactor containing 1 L of the SD-Ura-Leu medium with 2% raffinose (w/v) to have the final OD_600_ concentration of 0.2 OD/mL. Galactose induction was conducted after 10 h of cultivation with a final galactose concentration of 0.5% (w/v). The cultivation continued until a total cultivation time of 48 h was reached. 10 mL of culture was collected for acid phosphatase assay and chitin measurement and stored at −20 °C. The biomass was collected by centrifuging at 8000 rcf for 5 min and was washed twice with water before lyophilizing. The collected biomass was used for chitin extraction.

For the coproduction of storage lipids and chitin, yeast strains YAN45 and YAN46 were used. The first preculture was grown in 5 mL of the SD-Ura medium with 2% glucose (w/v) for 2 days at 30 °C and 220 rpm before transferring into 45 mL of the same medium to start the second preculture step in a 250 mL shake flask and incubated for 1 day under the same conditions. An appropriate volume of the second preculture was then added to the bioreactor containing 1 L of the SD-Ura medium with 2% glucose (w/v) to have the final OD_600_ concentration of 0.2 OD/mL. After 24 h of cultivation, the culture media were aseptically collected. The medium was exchanged by centrifuging the culture at 8000 rcf for 5 min to collect the yeast cells, before resuspending the pellet in 1 L of the fresh YCB-Ura medium and 250 mL of YCB induction solution lacking the sodium phosphate buffer. The resuspended cells were reintroduced into the bioreactors, and the cultivation continued for 48 h under the same conditions. 10 mL of culture was collected for chitin measurement and stored at −20 °C. The biomass was collected by centrifuging at 8000 rcf for 5 min and was washed twice with water before lyophilizing. The collected biomass was used for chitin extraction and lipid analysis by using GC-MS.

### 4.5 Chitin Measurement

Chitin content in the yeast cell wall was measured based on the staining method developed by Stagoj et al. (2004) with some modifications.^42^ An appropriate volume of culture equal to 2 × 10^7^ cells was collected by centrifugation at 14000 rpm for 3 min. The supernatant was removed, and the cell pellet was mixed with 100 μL of staining solution containing 2% glucose, 10 mM Na-HEPES pH 7.2, and 25 μM Calcofluor White M2R. The mixture was then incubated with shaking at 1000 rpm and 30 °C for 45 min. After that, the mixture was centrifuged at 14000 rpm for 3 min to remove the supernatant. The stained cell pellet was washed once with 100 μL of TE buffer (10 mM Tris-HCl, pH 8.0, 10 mM EDTA) to remove any excess staining solution. The pellet was then resuspended in 100 μL of TE buffer and transferred to a 96-well plate. The fluorescence signal was measured using a Synergy H1Microplate Reader with the following setup: excitation filter of 360/40 nm and emission filter of 460/40 nm; bottom read with 35% gain; read speed: normal; delay 100 ms; data point: 10; read height: 7 mm. A blank sample was prepared as 100 μL of TE buffer. The chitin content per 2 × 10^7^ cells (specific chitin content) of each sample was corrected by subtracting the value of the blank and is represented as relative fluorescence units (RFU). The total chitin yield is represented as volumetric chitin content (RFU/ml) and was calculated by multiplying the specific chitin content with the cell concentration counted using a hemocytometer. We verified the linearity of Calcofluor White M2R signal and chitin concentration using chitin nanocrystals (Figure S1).

### 4.6 Chitin Extraction

Chitin was extracted via an alkali treatment from the lyophilized biomass. The biomass was extracted at a w/v ratio of 1:40 in 1 M NaOH for 30 min at 121 °C. The chitin pellet was collected via centrifugation at 3900 rpm at room temperature for 5 min and washed intensively with distilled water and 95% ethanol until a neutral pH was reached before lyophilizing for 2 days. The extracted and dried crude chitin was weighed.

### 4.7 Fourier-Transform Infrared Spectroscopy (FT-IR)

Infrared spectra of chitin samples were collected with a PerkinElmer Spectrum 2 FT-IR spectrometer using an attenuated total reflection (ATR) sampling. The range of the FT-IR analysis was from 700 to 4000 cm^–1^ with 64 scans and a resolution of 4 cm^–1^. The data were analyzed using PerkinElmer Spectrum IR version 10.6.2. Chitin purchased from Sigma-Aldrich was used as a standard.

### 4.8 Acid Phosphatase Assay

Acid phosphatase (AP) assay was carried out following the protocol described in Frey and Aebi, 2015.^43^ 1 mL of culture was collected and centrifuged at 3900 rpm for 5 min. The supernatant was transferred into a fresh Eppendorf tube. 20 μL of cleared culture supernatant or the culture medium for blanks were dispensed in triplicates into the wells of a pre-warmed 96-well plate at 30 °C. The reaction was started by adding 100 μL of 20 mM 4-nitrophenyl phosphate dissolved in 0.1 M sodium acetate buffer (pH, 4.2) to each well. The plate was then incubated at 30 °C with the lid on for 30 min. The reaction was stopped by adding 200 μL of 2 M Na_2_CO_3_ to each well. Total AP activity (ΔA_450_) was measured as the absorbance at 405 nm using an Eon Microplate Spectrophotometer and normalized with a blank signal. AP activity per cell (Δ*A*_405_/10^7^cells) was calculated by dividing the total AP activity by the cell concentration counted using a hemocytometer.

### 4.9 Lipid Staining

The lipid content of the yeast was monitored based on a previously developed staining method from Miranda et al. (2020) with some modifications.^44^ An appropriate volume of culture equal to 2 × 10^7^ cells was collected and centrifuged at 14000 rpm for 3 min. The pellet was resuspended in 950 μL of PBS buffer with 5% DMSO (pH 7.4) in a 1.5 mL tube. A Nile Red stock solution was prepared freshly for every experiment by dissolving 1 mg of Nile Red in 1 mL of DMSO. The Nile Red stock solution was diluted 10 times using DMSO to obtain the Nile Red working solution of 100 μg/mL. 50 μL of the Nile Red working solution were added, and the cell suspension was mixed thoroughly by vortexing. In a dark environment, 250 μL of the suspension (5 × 10^6^ cells) were transferred, in triplicates, to black 96-well plates, and the fluorescence intensity was read immediately in a Synergy H1Microplate Reader with the following setup: excitation at 488 nm, emission at 585 nm, kinetic readings for 30 min with 1 min interval, and continuous orbital shaking. Blank samples were prepared by mixing 950 μL of PBS buffer with 5% DMSO (pH 7.4) and 50 μL of the Nile Red working solution. Yeast cells’ autofluorescence was measured by substituting the Nile Red working solution with 50 μL of PBS buffer with 5% DMSO (pH 7.4). After the kinetic readings, maximal emission values were determined. Specific lipid content (RFU), representing the lipid content of 5 × 10^6^ cells, was the maximal fluorescence readout corrected by subtracting the mean of blanks and the cells’ autofluorescence. The total lipid yield is represented as the volumetric lipid content (RFU/mL) and was calculated by multiplying the specific lipid content with the cell concentration counted using a hemocytometer.

### 4.10 Lipid Analysis

Lipid analysis was done according to the protocol described by Suutari et al., 1990.^45^ A 60 mg portion of dried biomass per sample was used for the analysis, and heptadecanoic acid methyl ester (Sigma) was added as an internal standard before the biomass was suspended in a saponification reagent. Gas chromatography–mass spectrometry (GC-MS) was used to analyze the lipid composition and concentration. The major fatty acids were identified from their GC-MS peak retention times relative to those of the internal standard. GC-MS was done using a Shimadzu GC-MS with Optic 4. The GC conditions were as follows: HP1MS (60 m × 0.25 mm × 0.25 μm) column; carrier gas helium, column flow-rate approximately 0.22 mL min^–1^; total hydrogen flow rate to the detector 5.4 mL min^–1^; linear velocity 12.5 cm/s; septum purge flow rate 3 mL min^–1^; split ratio 1:10; column inlet pressure 20 kPa; injector temperature 250 °C; detector temperature 250 °C; oven temperature was programmed from 120 to 250 °C at the rate of 10 °C min^–1^. The MS conditions were as follows: Ion Source Temp 200 °C; Interface Temp 250 °C; solvent cut time 4 min; start time 5 min; end time 25 min; acquisition mode scan; event time 0.3 s; scan speed 1666; start *m*/*z* 35; end *m*/*z* 500. Data analysis was performed on a GC-MS solution version 4.53SP1.

### 4.11 Fluorescence Microscopy

Fluorescence microscopy was used to visualize the intracellular lipid droplets and chitin in the cell walls of yeast cells. Calcofluor White M2R was used to stain chitin in the yeast cell wall. The staining protocol followed exactly the chitin measurement protocol mentioned above, except in the last step, in which the stained cells were fixed by resuspension in 100 μL of 3.75% formalin (v/v) solution and incubation in the dark at room temperature for 15 min. The cell suspension was then centrifuged at 14000 rpm for 3 min, and the pellet was washed twice with PBS buffer (pH 7.4) before resuspending in 100 μL of PBS buffer (pH 7.4).

The lipid droplets were stained using Nile Red, following the procedure for lipid content measurements described above, with the following modifications. After the addition of the staining solution, the cells were incubated in the dark at room temperature for 10 min before collecting the pellet by centrifugation at 14000 rpm for 3 min. The pellet was washed twice with PBS buffer (pH 7.4) before fixing with formalin, as described above.

Combined lipid droplets and chitin staining of cells were performed using both Nile Red and Calcofluor White M2R. Lipid droplets were stained as described above but without the fixation step, followed by the chitin staining protocol. Finally, the double-stained cells were fixed with formalin.

For imaging, 5 μL of stained and resuspended cell solution was pipetted onto a glass slide and covered with a coverslip. Fluorescence images were acquired using an Axio Observer Z1 microscope (Carl Zeiss, Germany) equipped with a 100×/1.4 Ph3 oil objective, a 1.6× tube lens, and a Photometrics Prime BSI camera. Calcofluor White was excited at 420 nm using an LED light source at 50% intensity. The emitted light was collected at a wavelength of 461–485 nm with a 100 ms exposure time. Nile Red was excited at 470 nm using an LED light source at 50% intensity. The emitted light was collected at a wavelength of 461–485 nm with a 150 ms camera exposure time. In addition, bright-field images were acquired with a Ph3 contrast objective.

## Supporting Information Available

The standard curve of chitin nanocrystal versus Calcofluor White fluorescence intensity, the list of oligonucleotides, and the list of plasmids and yeast strains used in this study (PDF)
