# Supplementary material for: Enhancing Chitin Production as a Fermentation Byproduct through a Genetic Toolbox That Activates the Cell Wall Integrity Response
Source: ACS Synth Biol. 2025 Jan 6;14(1):113–28. doi: 10.1021/acssynbio.4c00436 (PMC11744920; doi:10.1021/acssynbio.4c00436)
Supplement: Supplementary file 1 — sb4c00436_si_001.pdf [file sb4c00436_si_001.pdf]

Supplement information

Enhancing Chitin Production as a Fermentation Byproduct through a Genetic Toolbox That Activates the Cell Wall Integrity Response

An Nguyen[1], Isabell Tunn[1], Merja Penttilä[1], Alexander D. Frey[1*]

[1]Aalto University, Department of Bioproducts and Biosystems, 02150 Espoo, Finland

Corresponding authors' e-mail: alexander.frey@aalto.fi

Table S1. List of oligonucleotides

Primer's name	Primer's sequence	Function
OAF70	5' - AACCCGGGATGTCACAACAAGTTGGTAAC -3'	*RHO1* forward (fw)
OAF71	5'- AACTCGAGCTATAACAAGACACACTTCTTC -3'	*RHO1* reverse (rv)
OAF72	5' - AACCCGGGATGAGTTTTTCACAATTGGAGC - 3'	*PKC1* fw
OAF80	5'- AAAGTCGACTCATAAATCCAAATCATCTGGC -3'	*PKC1 rv*
OAF74	5'- TGGGATACCGCTGGTCACGAAGATTATGATAG - 3'	Point mutation *RHO1 fw*
OAF75	5'- CTATCATAATCTTCGTGACCAGCGGTATCCCA - 3'	Point mutation *RHO1 rv*
OAF76	5' - GGTGGACTACATGCGCATGGTGCTATTATC - 3'	Point mutation *PKC1 fw*
OAF77	5' - GATAATAGCACCATGCGCATGTAGTCCACC -3'	Point mutation *PKC1 rv*

Table S2. List of yeast strains

Strain	Relevant genotype	Ref
BY4742	*MATα his3Δ1 leu2Δ0 lys2Δ0 ura3Δ0*	Euroscarf
Δtgl3 Δtgl4	*MATα; ura3Δ0; leu2Δ0; his3Δ1; lys2Δ0; YMR313c::kanMX4; Δtgl4::Nat*	[1]
YAN61	BY4742 containing pRS416-*GPD*	This study
YAN55	BY4742 containing pAN33	This study
YAN15	BY4742 containing pAN17	This study
YAN18	BY4742 containing pAN20	This study
YAN54	BY4742 containing pAN32	This study
YAN14	BY4742 containing pAN16	This study
YAN17	BY4742 containing pAN19	This study
YAN13	BY4742 containing pAN15	This study
YAN56	BY4742 containing pAN31	This study
YAN16	BY4742 containing pAN18	This study
YAN19	BY4742 containing pAN21	This study
YAN57	BY4742 containing pAN27	This study
YAN58	BY4742 containing pAN28	This study
YAN59	BY4742 containing pAN29	This study
YAN60	BY4742 containing pAN30	This study
YAN45	*ΔTgl3ΔTgl4* containing pAN28	This study
YAN46	*ΔTgl3ΔTgl4* containing pJR025	This study
YAN53	BY4742 containing pAX461 and pAN31	This study
YAN61	BY4742 containing pAX461 and pRS416-*GAL*	This study

Table S3. List of plasmids

Plasmid	Description	Ref
pRS416-*GPD*		[2]
pRS416-*TEF*		[2]
pRS416-*GAL*		[2]
pRS414		[2]
pJR025	Recreated based on Mascorro-Gallardo et al.	[3]
pAX461		[4]
pAN33	pRS416-*TEF-RHO1*	This study
pAN17	pRS416-*TEF-PKC1*	This study
pAN20	pRS416-*TEF-PKC1*^{R398A}	This study
pAN32	pRS416-*GPD-RHO1*	This study
pAN16	pRS416-*GPD-PKC1*	This study
pAN19	pRS416-*GPD-PKC1*^{R398A}	This study
pAN15	pRS416-*GAL-RHO1*	This study
pAN31	pRS416-*GAL-RHO1*^{Q68H}	This study
pAN18	pRS416-*GAL-PKC1*	This study
pAN21	pRS416-*GAL-PKC1*^{R398A}	This study
pAN27	pJR025-*RHO1*	This study
pAN28	pJR025-*RHO1*^{Q68H}	This study
pAN29	pJR025-*PKC1*	This study
pAN30	pJR025-*PKC1*^{R398A}	This study

Figure S1. Standard curve of Calcofluor White stained chitin nanocrystal and emitted fluorescence signal of Calcofluor White.

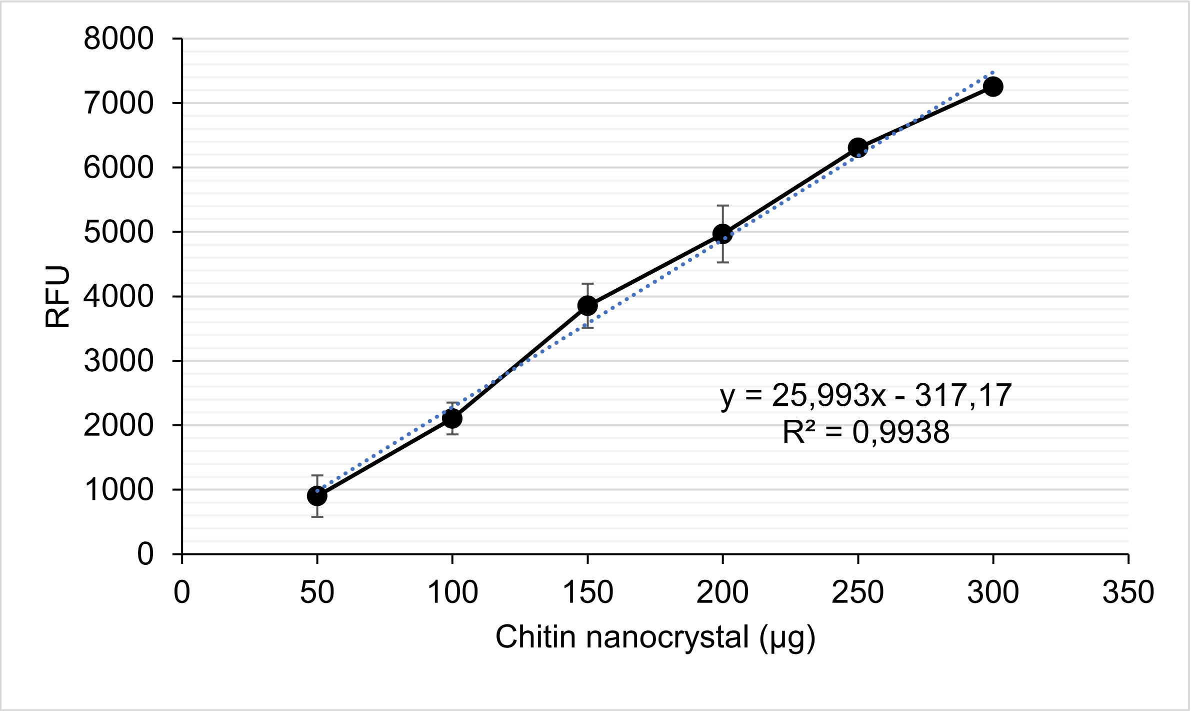

References

1. Hokkanen, S., S. Laakso,C.M. Senn, and A.D. Frey, *The trans-10,cis-12 conjugated linoleic acid increases triacylglycerol hydrolysis in yeast Saccharomyces cerevisiae.* Journal of Applied Microbiology, 2017. **123**(1): p. 185-193.
2. Mumberg, D., R. Müller, and M. Funk, *Yeast vectors for the controlled expression of heterologous proteins in different genetic backgrounds.* Gene, 1995. **156**(1): p. 119-22.
3. Mascorro-Gallardo, J.O., A.A. Covarrubias, and R. Gaxiola, *Construction of a CUP1 promoter-based vector to modulate gene expression in Saccharomyces cerevisiae.* Gene, 1996. **172**(1): p. 169-170.
4. Frey, A.D. and M. Aebi, *An enzyme-based screening system for the rapid assessment of protein N-glycosylation efficiency in yeast.* Glycobiology, 2015. **25**(3): p. 252-7.
